# AdipoR2 inhibits human glioblastoma cell growth through the AMPK/mTOR pathway

**DOI:** 10.1186/s40001-021-00496-9

**Published:** 2021-03-22

**Authors:** Chen Jie, Wang Xuan, Han-Dong Feng, Ding-Mao Hua, Wang Bo, Sun Fei, Zhang Hao

**Affiliations:** 1 Department of Neurosurgery, XuZhou Central Hospital, Xuzhou, China; 2 Xuzhou First People’s Hospital, The Affiliated Hospital of China University of Mining and Technology, Xuzhou, China

**Keywords:** AdipoR2, AMPK/mTOR, Antiproliferation, Glioblastoma cell

## Abstract

**Background:**

AdipoR2, which belongs to the seven-transmembrane-domain receptor family, has been shown to play an important role in the development of human tumours, but the underlying mechanisms are poorly understood. In this study, we found that AdipoR2 expression correlates with glioma grade. In addition, we also investigated the mechanisms behind the antiproliferative effects of AdipoR2 in U251 cells (a human glioma cell line) using colony formation and WST-8 growth assays.

**Methods:**

The U251 cell line was cultured in vitro. Western blotting was used to detect the expression of relevant proteins. Quantitative RT-PCR was used to detect AdipoR1 and AdipoR2 expression. Flow cytometry was used to detect cell cycle assay results. The gene expression profiles of glioma samples from the CGGA database were analysed by MATLAB and GSEA software.

**Results:**

The AMPK/mTOR pathway plays a central role in the regulation of cell proliferation, differentiation and migration and may promote tumorigenesis. Therefore, we can control cancer progression by modulating the AMPK/mTOR pathway. However, there is no information on the relationship between AdipoR and AMPK/mTOR in central nervous system tumours such as GBM. In this study. We found 648 upregulated genes and 436 downregulated genes correlated with AdipoR2 expression in 158 glioma samples. GSEA suggested that AdipoR2 is a cell cycle-associated gene. The results of the flow cytometry analysis indicated that AdipoR2 induced G0/G1 cell cycle arrest in U251 cells. Furthermore, we identified the AMPK/mTOR signalling axis to be involved in AdipoR2-induced cell cycle arrest.

**Conclusions:**

Our results suggest that AdipoR2 may represent a novel endogenous negative regulator of GBM cell proliferation. These findings also suggest that AdipoR2 may be a promising therapeutic target in GBM patients.

## Introduction

Glioblastoma (GBM) is a highly malignant and lethal cancer of the central nervous system. Recurrence is systematic, and prognosis is often poor. Regardless of surgery combined with radiation therapy and chemotherapy, patients suffering from malignant glioma have a life span of 12–15 months after diagnosis [1, 2]. Adiponectin (Acrp30), a 30-kDa component C1q-related protein, is implicated in cancer development. Most of the biological effects of Acrp30 are mediated by its receptors, AdipoR1 and AdipoR2, which belong to the seven-transmembrane-domain receptor family and have been shown to have abnormal expression in various types of human cancer [3–5]. Accumulating evidence suggests that Acrp30 has also a potential chemo-preventive role in carcinogenesis. Although the expression of AdipoR1 and AdipoR2 was previously observed in human cancer tissues, there are no clear indications about the presence of these receptors in human brain tumours.

Adenosine monophosphate-activated protein kinase (AMPK) acts as an important ‘metabolic sensor’ that is activated by increases in the adenosine monophosphate (AMP)/adenosine triphosphate (ATP) ratio and/or adenosine diphosphate (ADP)/ATP ratio [6]. Since it plays an important role in the regulation of energy homeostasis, AMPK is responsible for cancer cell proliferation and apoptosis. Therefore, targeting AMPK can induce apoptosis and inhibit cell proliferation [7]. Phosphorylated AMPK suppresses the mammalian target of rapamycin (mTOR) signalling pathway [8], which plays a central role in the regulation of cell proliferation, differentiation and migration [9, 10] and may promote tumorigenesis [6, 11]. Therefore, we can control cancer progression by modulating the AMPK/mTOR pathway [7, 12, 13]. Sugiyama et al. [14] reported that adiponectin inhibits colorectal cancer cell growth through the AMPK/mTOR pathway. However, there is no information on the relationship between AdipoR and AMPK/mTOR in central nervous system tumours such as GBM.

Therefore, there is an urgent need to determine the molecular mechanism involved in the pathogenesis of GBM and explore novel therapeutic strategies to treat this devastating disease. Considering the inhibitory role of AdipoRs in different tumours, we hypothesized that AdipoRs would likely influence GBM growth through the AMPK/mTOR pathway. In this study, we found that the expression of AdipoR2 correlates with glioma grade, so we further investigated the biological effect of AdipoR2 overexpression in U251 human cell lines.

## Materials and methods

### Human tissue samples

Messenger RNA (mRNA) expression data for 158 glioma samples were downloaded from the Chinese Glioma Genome Atlas (CGGA) data portal (http://www.cgga.org.cn/portal.phpg). The 158 glioma samples included 48 astrocytomas (As), 13 oligodendrogliomas (Os), 8 anaplastic astrocytomas (AAs), 10 anaplastic oligodendrogliomas (AOs), 15 anaplastic oligoastrocytomas (AOAs) and 64 glioblastoma multiforme tumours (GBMs). Tissue samples were obtained from the Department of Neurosurgery in Xuzhou Central Hospital from 2012 to 2015. Of these samples, three were normal brain tissues (NBTs), and 12 (3 grade II, 4 grade III and 5 grade IV) were glioma samples. The NBT samples were obtained from three patients who suffered severe brain trauma. This study was approved by the Medical Review Board of Xuzhou Central Hospital.

### Cell culture

The U251 cell line was purchased from the Chinese Academy of Sciences Cell Bank and cultured in Dulbecco’s modified Eagle’s medium (DMEM) supplemented with 10% foetal bovine serum (FBS), penicillin (100 U/mL) and streptomycin (100 μg/mL) (all from Invitrogen, Carlsbad, CA, USA) at 37 °C under a humidified atmosphere of 5% CO_2_. The U251 cell line was are authorized by the Chinese Academy of Sciences Cell Bank and Xuzhou Central Hospital for usement.

### Reagents and transfection

The recombinant plasmid for the pcDNA3.1 vector, which contains the ORF of human AdipoR2, was chemically synthesized and purified by GeneChem (Shanghai, China). The empty pcDNA3.1 vector was used as a negative control (NC). All plasmids were transfected into cells using Lipofectamine 2000 Transfection Reagent (Invitrogen, USA) according to the manufacturer’s instructions. The selective AMPK inhibitor compound C (iAMPK) was purchased from Calbiochem (La Jolla, CA, USA).

### Quantitative RT-PCR

RNA was extracted from tissues using TRIzol Reagent (Invitrogen). AdipoR1 and AdipoR2 qRT-PCR reactions were performed using Fermentas reverse transcription reagents and SYBR Green PCR Master Mix (Applied Biosystems) according to the manufacturer’s protocols. GAPDH was used for normalization. Relative gene expression was calculated via the 2^−△Ct^ method.

### WST-8 cell growth assay

U251 cells were seeded in 96-well culture plates at 2000 cells/well/100 µL. The cells were treated with AdipoR2 for 1–4 days. Then, tetrazolium monosodium salt WST-8 (Dojindo, Japan) was added (10 µL/well). After incubation for 2 h, the absorbance was determined using a microplate reader (Bio-Rad, USA) at a wavelength of 450 nm with the reference wavelength set at 630 nm.

### Colony formation assay

U251 cells were seeded in six-well plates and cultured overnight. Then, AdipoR2 or NC was transfected into the cells. After 48 h, 5 × 10^2^ treated and untreated cells were independently plated onto 60-mm tissue culture plates. After incubation for 2 weeks, visible colonies were fixed with 4% methanol for 30 min and stained with 0.1% crystal violet for 20 min. Colony-forming efficiency was calculated using the following equation: colony-forming efficiency = number of colonies/number of plated cells × 100%.

### Cell cycle assay

Forty-eight hours posttransfection, U251 cells were collected and fixed with 70% ethanol at − 20 °C overnight. DNA was stained by incubating cells in 50 mg/mL propidium iodide (PI) (Sigma-Aldrich, USA) and 10 mg/mL RNase A (Boehringer-Mannheim, Germany) for 1 h at room temperature. The cells were then analysed by FACScan (Becton-Dickinson, USA).

### Western blotting

Proteins were extracted in lysis buffer according to the manufacturer’s protocol. Lysates were separated by SDS-PAGE and transferred to a nitrocellulose membrane (Bio-Rad, USA). The membranes were incubated in blocking buffer. The membranes were incubated at 4 °C overnight with primary antibodies against the following proteins: AdipoR2 (Abcam, USA), AMPK (Abcam, USA), p-AMPK (Abcam, USA), mTOR (Abcam, USA), p-mTOR (Abcam, USA), S6K (Abcam, USA), pS6K (Abcam, USA), S6P (Abcam, USA)), phosphorylated (Ser240/244) S6 ribosomal protein (pS6P) and GAPDH (CST, USA). Immunoreactivity was visualized with horseradish peroxidase-conjugated goat anti-rabbit antibody (Bioworld, USA). The protein bands were detected and imaged with a ChemiDocXRS^+^ Gel Imaging System (Bio-Rad, USA) and analysed by densitometric quantification using ImageJ software.

### Gene set enrichment analysis with AdipoR2 expression

The gene expression profiles of glioma samples from CGGA were analysed by GSEA [15]. Pearson’s correlation was used to analyse the relationship between AdipoR2 and all identified genes with MATLAB software (*P* < 0.001). GSEA (http://www.broadinstitute.org/gsea/) analysis was used to identify pathway gene sets that were correlated with the AdipoR2 expression profile. For GSEA, AdipoR2 expression was treated as a binary variable divided into low or high AdipoR2 expression, and the cut-off point was 50%. As a metric for ranking genes in the GSEA, the difference between the means of samples with low and high AdipoR2 expression was used, and the other parameters were set to their default values.

### Statistical analysis

Kaplan–Meier survival analysis was used to estimate survival distributions. The log-rank test was used to assess the statistical significance between stratified survival groups using GraphPad Prism. The *t* test was used to determine differences in each 2-group comparison. All statistical analyses were performed using MATLAB 2012, SPSS software for Windows, or GraphPad Prism (GraphPad Software). All data are presented as the means ± standard error. A 2-sided *P* value of < 0.05 was regarded as significant.

## Results

### AdipoR2 expression correlates with glioma grade

**Fig. 1:**
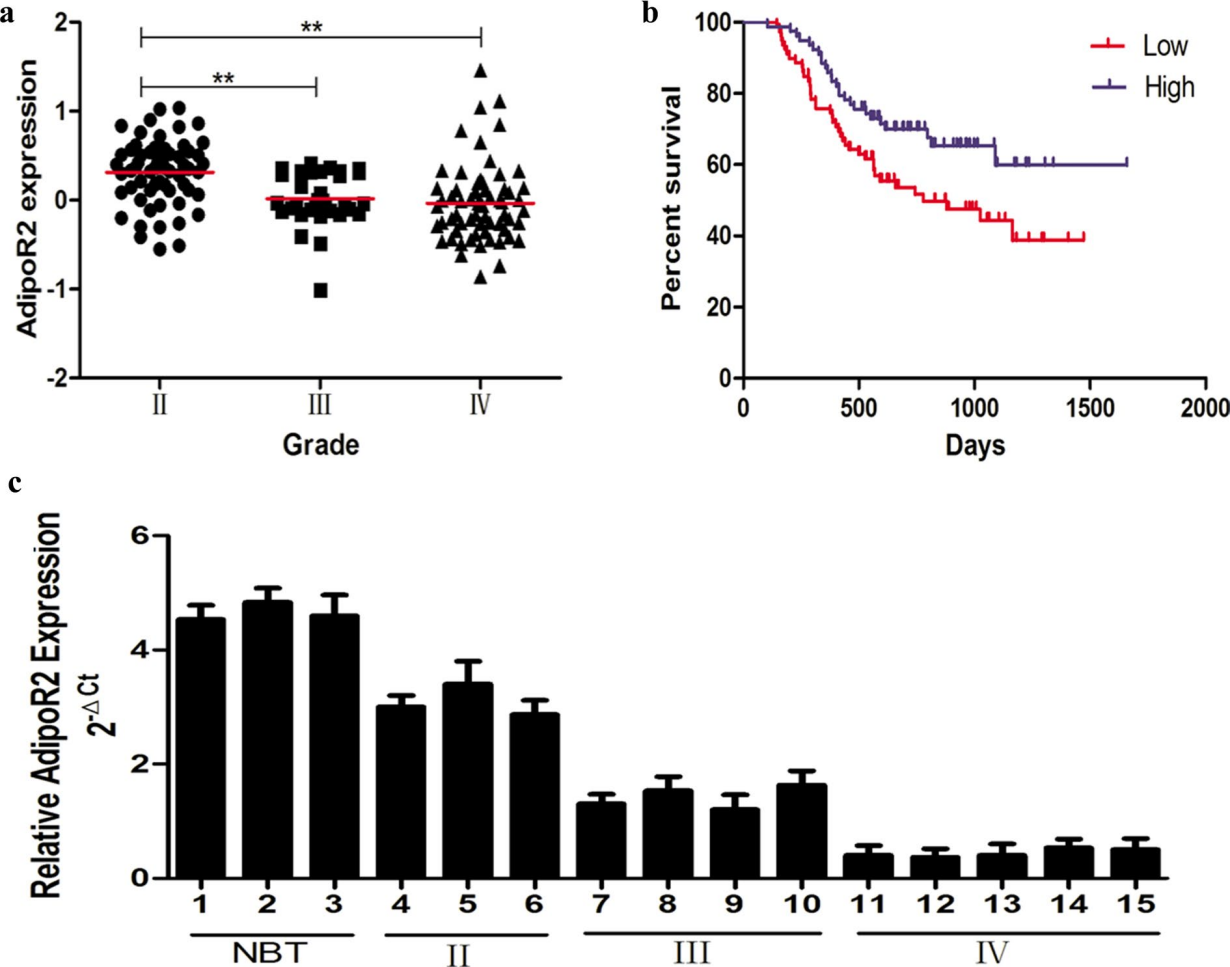
Clinical significance of AdipoR2 in glioma cases. **a** The levels of AdipoR2 were analysed in glioma tissues from the CGGA glioma database. **b** Kaplan–Meier survival curves for AdipoR2 expression in glioma tissues from the CGGA glioma database. **c** qRT-PCR analysis showed decreased AdipoR2 levels in high-grade glioma tissues compared with those in low-grade glioma tissues and normal brain tissues. **P < 0.01, *P < 0.05

First, we analysed AdipoR2 expression levels in whole genome gene profiling of 158 glioma tissue samples. AdipoR2 expression was significantly lower in grades III–IV than in grade II (Fig. 1a), but no significant relationship was observed between AdipoR1 expression and glioma grade (data not shown). As shown in Fig. 1b, we also investigated the prognostic value of AdipoR2 in the 158 glioma samples by Kaplan–Meier survival analysis. The results indicated that patients with high AdipoR2 expression had a longer mean overall survival (OS) than patients with low AdipoR2 expression. In addition, we also analysed 15 samples of varying grades of glioma and normal brain tissues, and the results were consistent with the CGGA database (Fig. 1c). These findings suggest that AdipoR2 may play an important role in glioma development.

### AdipoR2 inhibits U251 cell proliferation

**Fig. 2:**
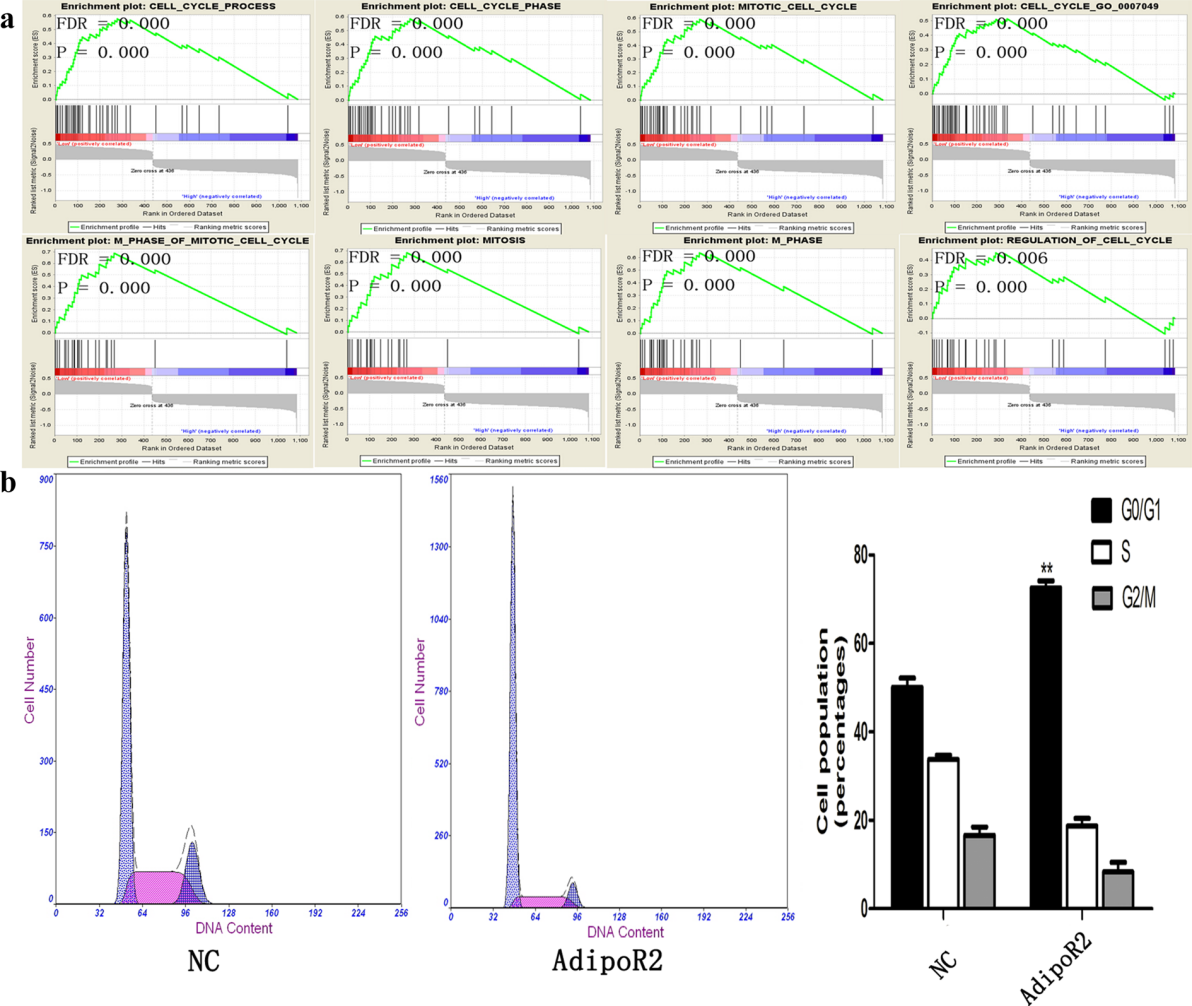
AdipoR2 is a cell cycle-associated gene. **a** GSEA of gene ontology showed that genes correlated with AdipoR2 in glioma patients were involved in cell cycle progression. **b** After treatment with AdipoR2, U251 cells were used for cell cycle analyses with propidium iodide staining and flow cytometry analysis. **P < 0.01, *P < 0.05

Next, to explore the effects of AdipoR2 on U251 cell proliferation, U251 cells were treated with AdipoR2 for 1, 2, 3, and 4 days. The proliferation of these AdipoR2-treated cancer cells was then assessed using WST-8 assays. As shown in Fig. 2b, AdipoR2 significantly inhibited cell proliferation in a time-dependent manner. In addition, we also used a colony formation assay to determine the role of AdipoR2 in glioma cell proliferation. As shown in Fig. 2c, the colony formation assay showed that the number of colonies in the experimental groups was obviously lower than that in the NC group.

### AdipoR2 is a cell cycle-associated gene

**Fig. 3:**
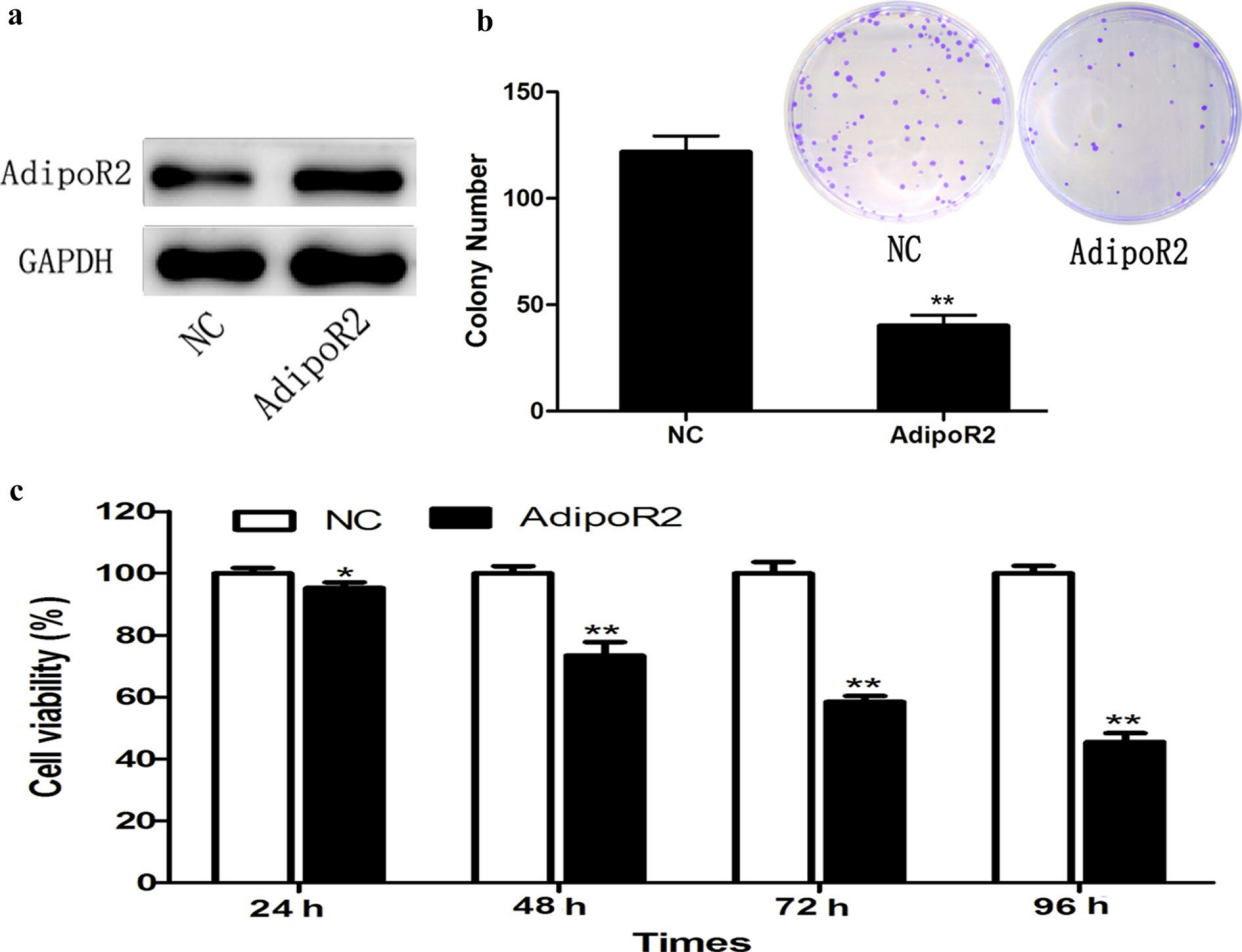
AdipoR2 inhibited U251 cell proliferation. **a** Western blot results for AdipoR2 protein levels in U251 cells treated with AdipoR2. **b** WST-8 assays were conducted on U251 cells after treatment with AdipoR2. **c** A colony formation assay was performed on U251 cells after treatment with AdipoR2. **P < 0.01, *P < 0.05

To identify the mechanism of AdipoR2 involvement in glioma, we first screened differentially expressed genes and found 648 upregulated genes and 436 downregulated genes related to AdipoR2 expression in the 158 glioma samples. These genes will be called “AdipoR2-associated genes”. GSEA was used to analyse the pathways that were differentially expressed between patients with high levels of AdipoR2 expression and those with low levels of AdipoR2 expression. GSEA revealed that AdipoR2 regulates genes primarily associated with cell cycle progression (Fig. 3a).

The GSEA results indicated that AdipoR2 is involved in the cell cycle. To provide evidence to support this result, we evaluated cell cycle distribution using flow cytometry. As shown in Fig. 3b, the cell cycle assay indicated that U251 cells in the AdipoR2 transfection group were notably arrested in the G0/G1 phase compared with those in the NC group.

### AdipoR2-induced G0/G1 arrest and antiproliferative effects occur in U251 cells via the AMPK/mTOR pathway

**Fig. 4:**
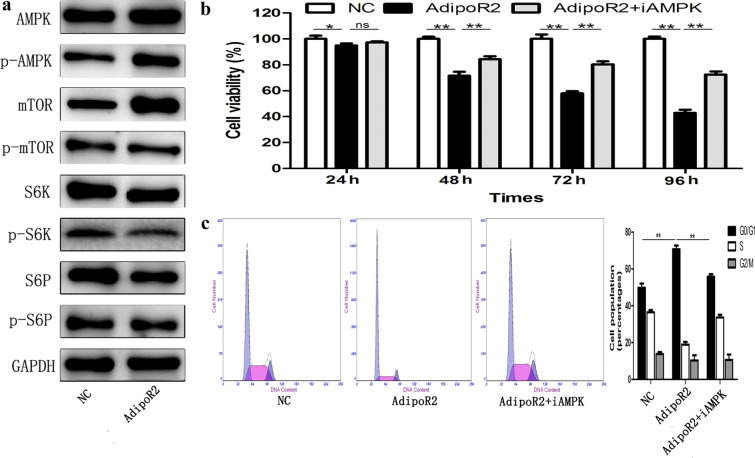
Evaluation of the involvement of the AMPK–mTOR signalling pathway in AdipoR2-induced G_0_/G_1_ arrest. **a** Western blot analysis of AMPK, p-AMPK, mTOR, p-mTOR, S6K, pS6K, S6P, and pS6P protein levels in U251 cells after treatment with AdipoR2. **b**, **c** Rescue experiment performed by introducing iAMPK into U251 cells in the presence or absence of AdipoR2. **P < 0.01, *P < 0.05

The effect of AdipoR2 on the AMPK/mTOR signalling pathway was examined. The Western blot analyses shown in Fig. 4a revealed that the expression levels of S6K, pS6P, AMPK and mTOR were not significantly affected by AdipoR2 overexpression, whereas the phosphorylation of S6K, pS6P, AMPK and mTOR was obviously affected (Fig. 4a). Next, we assessed whether overexpression of AdipoR2 induces cell cycle arrest and inhibits cell growth via the AMPK/mTOR pathway. First, the selective AMPK inhibitor compound C (iAMPK) was used. U251 cells were treated with iAMPK, and the AdipoR2 plasmid was subsequently transfected into all cells. The effects of AdipoR2 overexpression on cell cycle arrest in U251 cells were partially rescued by iAMPK (Fig. 4b). In addition, iAMPK treatment significantly rescued AdipoR2-induced inhibition of cell growth in a time-dependent manner (Fig. 4c). These results indicated that AdipoR2-induced cell growth inhibition is mediated by AMPK activation.

## Discussion

GBM is the most common and deadly primary brain tumour and displays rapid cell growth and resistance against apoptosis in adults [16]. Accumulating evidence has shown that the expression of AdipoRs (AdipoR1 and AdipoR2) can be observed in human cancer tissues [17]. Porcile et al. demonstrated that AdipoR1 and AdipoR2 are often coexpressed in GBM tissue, but the study did not compare the differences between AdipoR type (AdipoR1 and AdipoR2) and glioma grade [18]. In the present study, we identified a significant negative association between AdipoR2 expression and glioma grade, but no statistically significant associations were observed between AdipoR1 expression and glioma grade. In addition, AdipoR2 is downregulated in human gastric cancer and endometrial adenocarcinoma [4, 19]. Knockdown of AdipoR2 relieved the suppressive effects of adiponectin on the growth of colon cancer cells [5]. These studies demonstrated that AdipoR2 functions as a novel regulator of cell proliferation in various human cancers. Consistent with these results, we found that AdipoR2 inhibited glioma cell proliferation and induced G0/G1 arrest in U251 cells.

AMPK is a ubiquitous serine/threonine protein kinase that has been found to regulate cellular energy metabolism [6]. In addition, AMPK plays a significant role in cell proliferation. mTOR-dependent activation of the AMPK signalling pathway can control cell growth in all eukaryotes and is deregulated in most human cancers [20]. One mechanism by which AMPK controls mTOR is via direct phosphorylation of its substrates. The 70-kDa ribosomal proteins S6 kinase (S6K) and S6 ribosomal protein (S6P) are included in the signalling cascade downstream of mTOR. They are both activated via phosphorylation by mTOR [21]. Mounting evidence shows that dysregulation of the AMPK/mTOR signalling pathway is associated with a variety of cancers [12, 14]. In our study, we found that AdipoR2 inhibited glioma cell proliferation. However, the mechanisms through which AdipoR2 affects cancer cells have not been completely elucidated. AdipoR2, whose activation results in the modulation of different protein kinases, including AMPK [22], is involved in the Acrp30-mediated modulation of several metabolic processes, such as glucose and fatty acid metabolism [23]. Thus, there is a possibility that AdipoR2 inhibits human glioblastoma cell growth through the AMPK/mTOR pathway. In line with this hypothesis, we found that AdipoR2 downregulated the expression of p-AMPK. We also observed significant phosphorylation of mTOR, pS6K, and pS6P after treatment with AdipoR2. In addition, the selective AMPK inhibitor compound C (iAMPK) significantly blocked AdipoR2-induced cell cycle arrest in U251 cells. Taken together, these results led us to conclude that the AMPK/mTOR pathway is highly important for AdipoR2-induced anticancer effects.

Most importantly, we investigated the effect of AdipoR2 overexpression on glioma cell proliferation. The inhibitory effect of AdipoR2 was mediated by the AMPK-activated mTOR pathway. To our knowledge, this is also the first report to provide a basis for studying the implications of crosstalk between AdipoR2 and the AMPK–mTOR signalling pathway in GBM.

## Conclusions

We investigate the effect of AdipoR2 overexpression on the glioma cell proliferation. This inhibiting effect is mediated by AMPK-activated mTOR pathway. To our knowledge, this is also the first report to provide a rationale for the implication of cross-linking between AdipoR2 and AMPK–mTOR signalling pathway in GBM.

## Data Availability

All of the data and materials are available.
